# Supplementary material for: Characterization of Tetrathionate Hydrolase from Acidothermophilic Sulfur-Oxidizing Archaeon Metallosphaera cuprina Ar-4
Source: Int J Mol Sci. 2025 Feb 5;26(3):1338. doi: 10.3390/ijms26031338 (PMC11818568; doi:10.3390/ijms26031338)
Supplement: Supplementary file 1 [file ijms-26-01338-s001.zip › ijms-3436743-supplementary.pdf]

Supplemented Materials

Characterization of Tetrathionate Hydrolase from Acidothermophilic Sulfur-oxidizing Archaeon *Metallosphaera cuprina* Ar-4

Pei Wang[1,2#], Liang-Zhi Li[3#], Li-Jun Liu[4], Ya-Lin Qin[1,2], Xiu-Tong Li[1,2], Hua-Qun Yin[3], De-Feng-Li[1,2], Shuang-Jiang Liu[1,2]* and Cheng-Ying Jiang [1,2]*

[1]State Key Laboratory of Microbial Resources, Institute of Microbiology, Chinese Academy of Sciences, Beijing 100101, China
[2]University of Chinese Academy of Sciences, Beijing 100049, China
[3]School of Minerals Processing and Bioengineering, Key Laboratory of Biometallurgy of Ministry of Education, Central South University, Changsha 410083, China
[4]School of Basic Medical Science, Xi'an Medical University, Xi'an, Shaanxi 710021, China

*Corresponding author: Cheng-Ying Jiang & Shuang-Jiang Liu

E-mail: jiangcy@im.ac.cn & liusj@im.ac.cn

Running title: Tetrathionate hydrolase from *Metallosphaera cuprina* Ar-4

Keywords: *Metallosphaera cuprina*, tetrathionate hydrolase, localization, purification, kinetics of enzyme

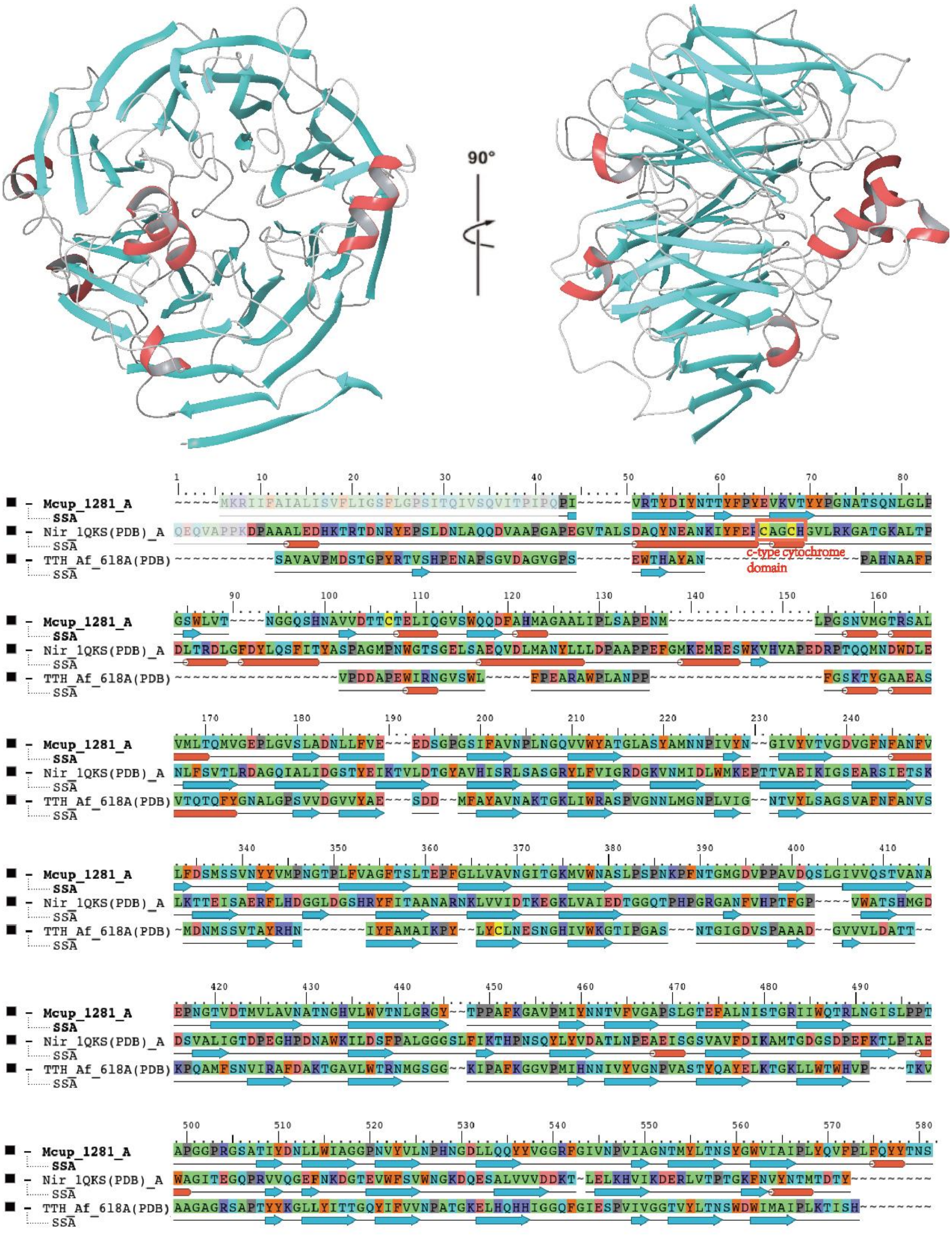

Figure S1. AlphaFold2-predicted structure of tetrathionate hydrolase (TTH) from *Metallosphaera cuprina* Ar-4 (top). The secondary structure of TTH_{Mc} monomer is colored in blue (β-strands) and red (α-helices), respectively. Multiple sequence alignments of TTH_{Mc} and closely related PDB entries (bottom).

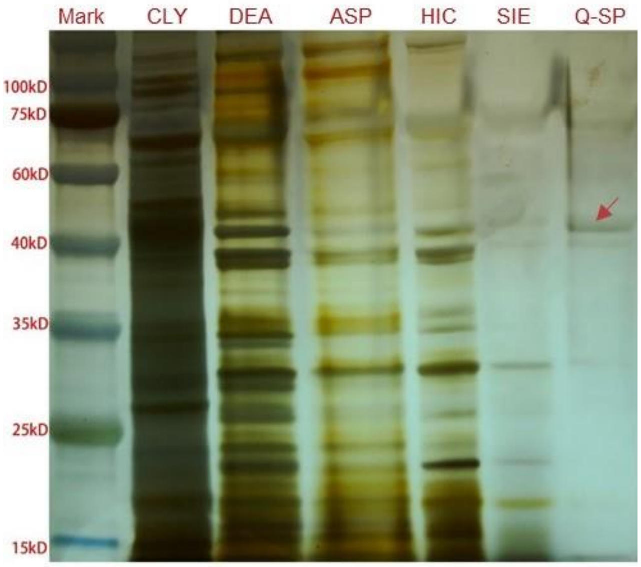

Figure S2. Silver-stained SDS gels of different purification steps of TTH_{Mc}; CLY, Cell lysate, DEA, Active fractions of DEAE anion exchange chromatography; ASP Ammonium sulfate precipitates; HIC, hydrophobic chromatography; SIE, size exclusion chromatography; Q-SP Q-sepharose chromatography; 30μL protein was loaded in each lane; Red arrow represented the TTH_{Mc} band.

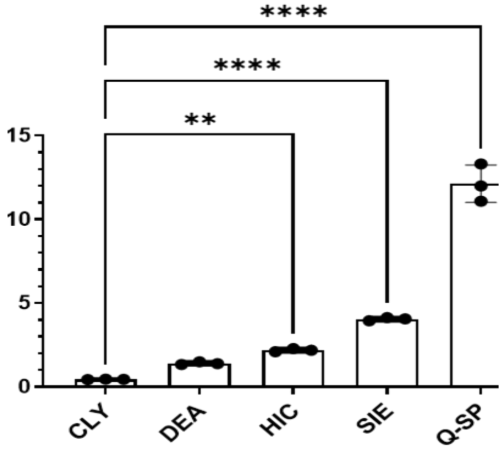

Figure S3. Specific activity for TTH of *M. cuprina* Ar-4 during the purified process; CLY, cytoplasm; DEA, active fractions of DEAE anion exchange chromatography; HIC, hydrophobic chromatography; SIE, size exclusion chromatography; Q-SP, Q-sepharose chromatography. Error bars represent SD of n=3 independent measurements. Significance was determined using one –way ANOVA analysis, ** $p < 0.01$, *** $p < 0.001$, **** $p < 0.0001$.

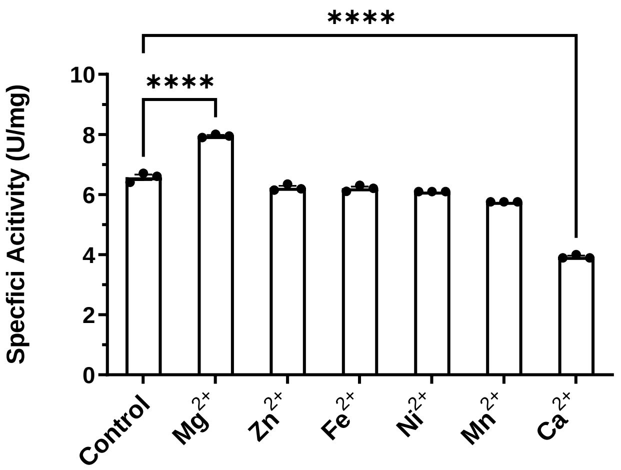

Figure S4. Effect of bivalent metal ion on the activity of the TTH from *M. cuprina* Ar-4. The concentrate of metal ion Mg, Zn, Fe, Ni, Mn, Ca are all 0.01mmol/L. Significance was determined using one –way ANOVA analysis, **** $p < 0.0001$.

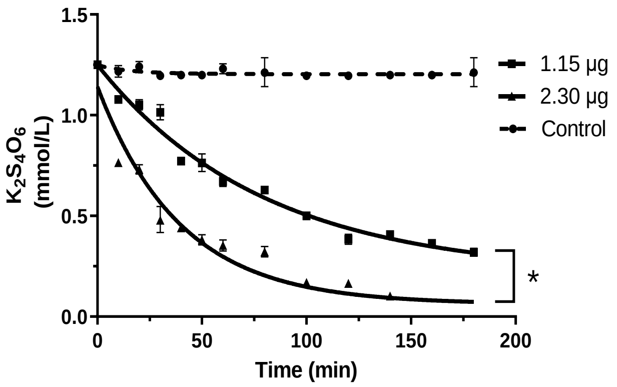

Figure S5. The reaction process of tetrathionate catalyzed by various concentrations of TTH_{Mc}; The dotted line represented reaction without TTH_{Mc} enzyme; The solid line with square represented reaction with 1.15 μg TTH_{Mc}; The solid line with triangle represented reaction with 2.30 μg TTH_{Mc}; Error bars represent SD of n=3 independent measurements. Significance was determined using one-way ANOVA analysis. * $p < 0.05$.

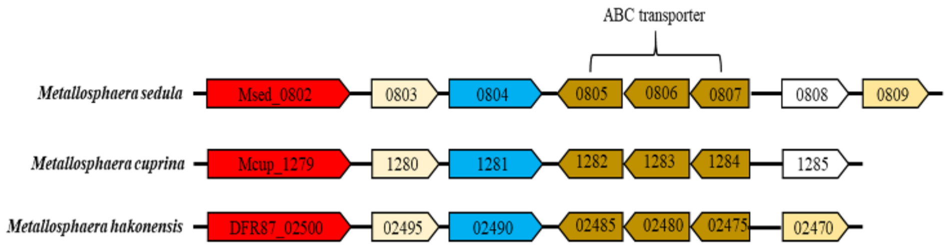

Figure S6. TTH clusters are conserved in *Metallophaera* spp.

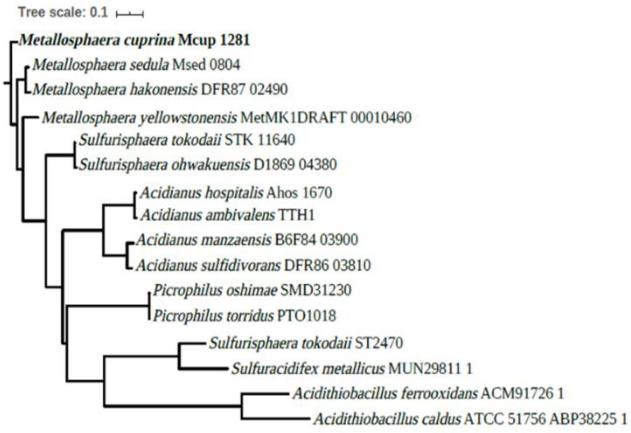

Figure S7. Phylogenetic relationship of TTHs among acidophilic archaea and bacteria.

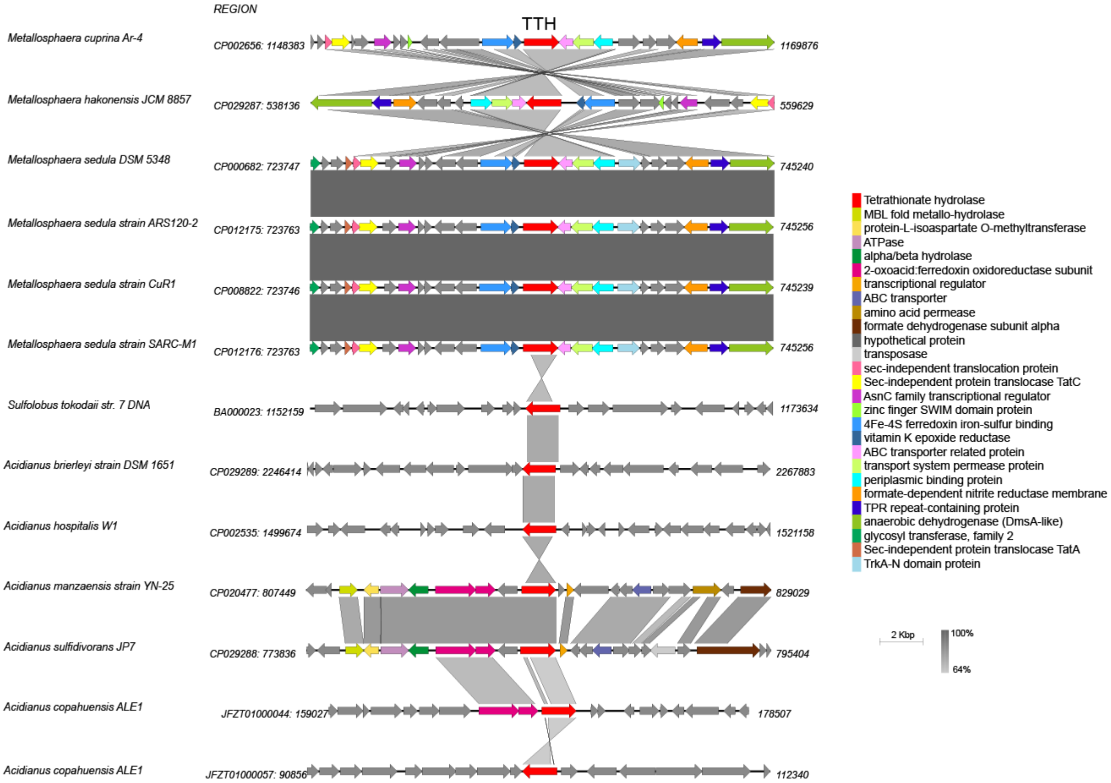

Figure S8. Genome context comparison of TTHs from Sulfolobaceae strains shows highly variable gene contexts.

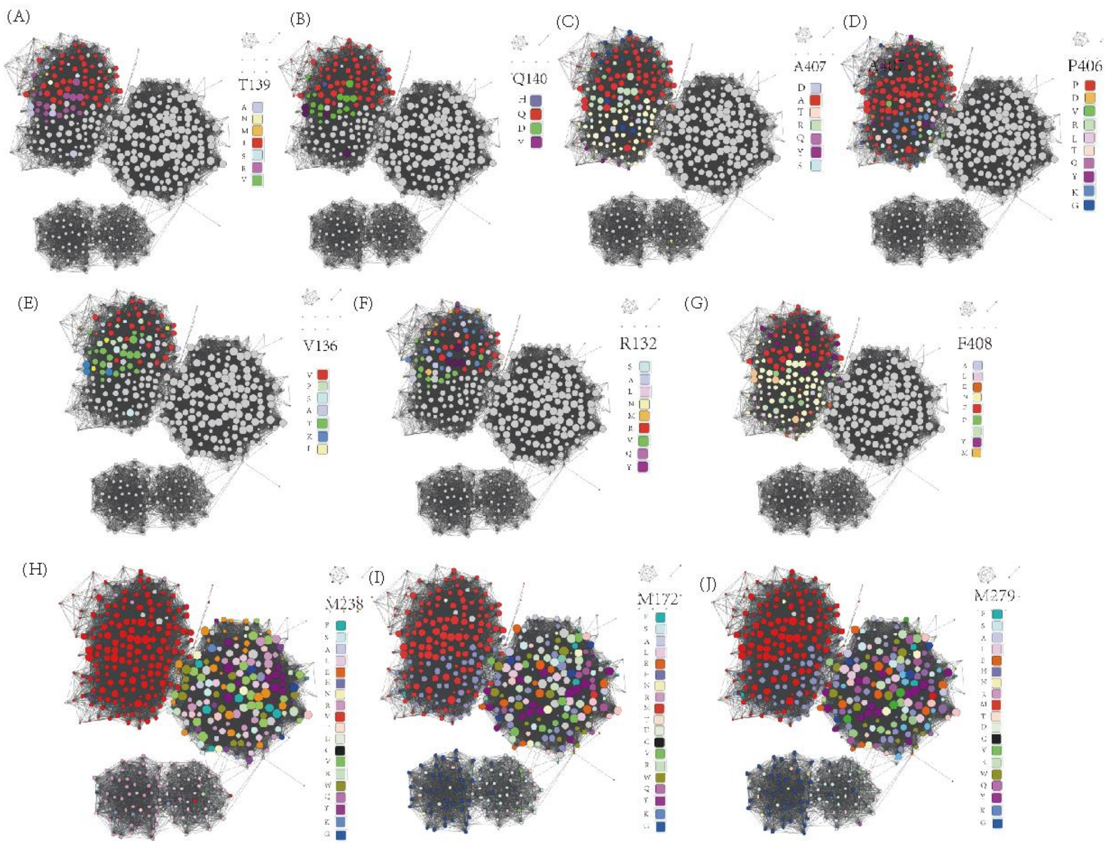

Figure S9. Sequence similarity network (SSN) built with TTH_{Mc} as the query colored by the type of the residues on the respective positions of TTH_{Mc} (A) T139; (B) Q140; (C) A407; (D) P406; (E) V136; (F) R132; (G) F408; (H) M238; (I) M172; (J) M279.

Table S1. The TTH total activity and percentages in differential cell fractions of whole cell

Cell components	Protein amount (mg)	Total activity (U)	Related activity (%)	Specific activity (U/mg)	Pyrophospahtase activity (U)
Periplasm	0.055 ± 0.009	0.39 ± 0.06	24.1	7.1 ± 0.1	1.36 ± 0.07
Cytoplasm	2.24 ± 0.67	1.17 ± 0.33	72.3	0.522 ± 0.010	113.03± 5.90
Membrane (pH3)	6.10 ± 1.21	0.059 ± 0.014	3.6	0.010 ± 0.000	0.27 ± 0.02
Membrane (pH7)	7.87 ± 0.19	0.068±0.002		0.009 ± 0.000	0.18 + 0.02
